# Intercomparison of Indexable Cutting Inserts’ Wear Progress and Chip Formation During Machining Hardened Steel AISI 4337 and Austenitic Stainless Steel AISI 316 L

**DOI:** 10.3390/ma17225418

**Published:** 2024-11-06

**Authors:** Karel Šramhauser, Pavel Kraus, František Špalek, Pavel Černý, Jean de Dieu Marcel Ufitikirezi, Tomáš Zoubek, Miroslav Strob, Yevhen Kononets, Pavel Kříž, Vladimír Vochozka

**Affiliations:** 1 Department of Technology and Cybernetics, Faculty of Agriculture and Technology, University of South Bohemia in České Budějovice, 370 05 České Budějovice, Czech Republic; fspalek@fzt.jcu.cz (F.Š.); pcerny@pf.jcu.cz (P.Č.); ufitikirezi@fzt.jcu.cz (J.d.D.M.U.); zoubek@fzt.jcu.cz (T.Z.); miross00@fzt.jcu.cz (M.S.); kononets@fzt.jcu.cz (Y.K.); kriz@pf.jcu.cz (P.K.); 2 Institute of Technology and Materials, Faculty of Mechanical Engineering, Jan Evangelista Purkyně University in Ústí nad Labem, 400 96 Ústí nad Labem, Czech Republic; pavel.kraus@ujep.cz; 3 Department of Applied Physics and Technology, Faculty of Education, University of South Bohemia in České Budějovice, 371 15 České Budějovice, Czech Republic; vvochozka@pf.jcu.cz

**Keywords:** indexable cutting insert, wear, hardened steel, stainless steel, chip production

## Abstract

This article deals with a mutual comparison of indexable cutting inserts of the CNMG 120408 type from two different manufacturers during the machining of hardened steel AISI 4337 and austenitic stainless steel AISI 316 L. The main goal is to analyse the different wear processes depending on the difference in the manufacturer’s design and also depending on the properties of the different machined materials. The progress of the wear of the main spine of the tool, the types of wear and the service life of the cutting edge were monitored, with the achievement of the critical value VB_max_ = 300 µm being the standard. In addition to the wear of the inserts, the production of chips was monitored in terms of their shape, average size and number of chips per 100 g of chips produced. In order to understand the relationships arising from the obtained data, an SEM equipped with an elemental analyser was used to analyse the coating layers and the substrate of the unworn inserts and the types of wear and the intensity of the surface damage of the worn inserts. A several-fold difference in the lifetime of the cutting edge was found, both in terms of design and in terms of the selected machined material, while in both cases the cutting edge with Al_2_O_3_ and TiCN layers of half thickness achieved a better result in liveness. From the point of view of chip formation, very similar results in shape and average length were observed despite the different designs of chip breakers. Cutting inserts with half the thickness of the coating layers achieved longer cutting edge life in the non-primary material application compared to the target workpiece material. At the same time, it was observed that a thinner coating layer has a positive effect on chip formation in terms of its length and shape.

## 1. Introduction

In the contemporary manufacturing sector, businesses are looking to implement a uniform carbide cutting element system that facilitates the easy exchanging of cutting components [1,2]. The geometric, physical and chemical properties of cutting elements directly influence their capabilities and performance [3,4,5]. These characteristics of each individual cutting element play a crucial role in influencing its performance when engaging with the workpiece and the surrounding conditions. Moreover, every cutting material exhibits an inherent inclination to alter cutting parameters, as well as to experience abrasive and adhesive wear as time progresses [3,6,7]. An inappropriate cutting tool for a specific manufacturing scenario possesses the potential to diminish the quality of the surface finish and escalate the rates of production defects. Moreover, it can considerably curtail the lifespan of the tool and augment the consumption of process fluid. Additionally, it leads to an upsurge in the expenses associated with the disposal of production waste [8,9,10].

The advancement of cutting materials has significantly improved since the introduction of the electric arc furnace. A key benefit of the arc furnace is its capability to melt tough materials like carbides. Carbides that are sintered exhibit superior bending strength, Young’s modulus and fracture toughness compared to other cutting materials, making them ideal for high-speed machining and intermittent cutting in challenging environments [11,12]. The disadvantage is lower thermochemical and thermomechanical stability; for example, dry turning iron-based superalloy GH2132 limits the use at higher cutting speeds at >30 m·min^−1^ and higher than 250 m·min^−1^ when turning AISI 4340 steel (220 HV) [13,14]. Sintered carbide is a heterogeneous material consisting of solid carbide particles in a metal bond. For practical purposes, mainly hexagonal tungsten carbide (WC) and cubic carbides of titanium (TiC), tantalum (TaC) and niobium (NbC) are used in the manufacture of cutting tools [15,16,17]. These carbide particles improve strength, hardness and thermomechanical stability. Their geometrical form is of spherical shape and the size typically ranges from 0.1 to 19 µm [12]. The cobalt (Co) bond is the most prevalent method of anchoring carbide particles, typically comprising up to 13%, with the volume of the bond ranging from 5 to 40% of the total volume [18,19,20]. In order to enhance cutting capabilities, coatings are applied either across the entire surface or on specific functional areas. These coatings are typically administered in multiple layers, each possessing unique properties aimed at bolstering the resistance to mechanical wear of cutting inserts. Consequently, this process can significantly prolong the lifespan of these inserts by a factor of 2 to 10 times [21,22].

Sintered carbide cutting tools offer a distinct benefit through their well-established disposal and recycling process, which involves transforming them into atomic particles, purifying and separating them, and ultimately crystallising them into primary powders [23,24,25]. In machining, along with dulling of the cutting tool, the primary outcome is the removal of material from the workpiece in the form of chips. These metal chips are characterised by their dimensions and configuration, with particular emphasis on their length [26,27]. The assessment of metal chips in terms of both quantity and characteristics is frequently discussed within the realm of waste management. When dealing with long, uninterrupted shavings, the operational area of the machinery becomes filled rapidly, necessitating frequent emptying. The presence of substantial amounts of waste in the shape of long, continuous chips results in the occupation of excessive storage space and complicates subsequent machining processes. When elementary or fine mesh shavings are generated, storage space efficiency is increased, waste removal frequency in the production area is decreased and the recycling process operates at a higher level of performance [28,29,30]. The characteristics of a metal swarf, including its shape and length, are influenced by various factors. These factors include the properties of the material being machined, the specific cutting conditions employed, the application of process fluids at the cutting points, the geometry of the cutting tool, the properties of the cutting material and the type of coating used on the cutting tool [31]. The description and classification of the shape, size and direction of the chip emerging from the cutting point are covered in ISO 3685:1993 [32]. Numerous research studies have explored the enhancement of the cutting process through the utilisation of the Taguchi method and ANOVA (analysis of variance) [33,34,35]. When assessing cutting tool wear, the chip formation factor plays a crucial role in monitoring both the wear process and the overall machining process [36].

Machining is inherently a process that is influenced by many factors, each of which influences the outcome of the job. This result again consists of a set of goals that we want to achieve when creating the workpiece in terms of its surface properties, level of residual stresses, ideal handling of waste material, etc. The data analysed as part of this research demonstrate the influence of subtle manufacturing nuances of interchangeable inserts of the same type according to ISO 1832:2017 on their durability, course and rate of wear depending on the properties of the test materials [37]. At the same time, this article compares the chips produced from individual cutting processes and thus also adds to the factor of wear of the cutting tool the aspect of the influence of the mentioned production nuances, which are not subject to standardised marking, on the formation of chips in terms of their length and shape, which are important parameters when planning the cutting process and subsequent handling with waste material.

## 2. Materials and Methods

The aim of the experiment was to compare the CNMG 120408 type of indexable inserts in terms of their service life (specifically the progression of their wear) and their suitability for chip formation. For this purpose, a procedure was designed to ensure the comparability of the results obtained from the data. This experiment aimed to demonstrate the wear mechanisms of cutting tools and chip formation in relation to the selected cutting parameters as well as differences in the design of the specific tools. Additionally, the experiment incorporated the factor of the material properties of the workpieces, as demonstrated by the different types of steel used. The experiment was conducted in collaboration between the Faculty of Agriculture and Technology at the University of South Bohemia in České Budějovice and the Faculty of Mechanical Engineering at the Jan Evangelista Purkyně University, within the framework of research into tool wear mechanisms. The following sections describe the test materials, the selected indexable cutting inserts and the methodological approach, including the measuring equipment used.

### 2.1. Evaluated Cutting Inserts

**Figure 1:**
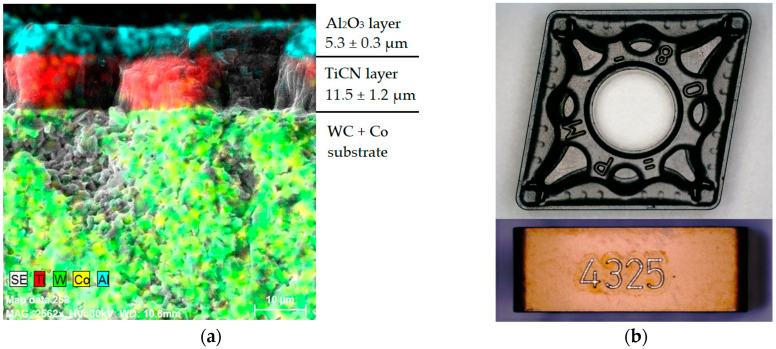
Investigated CNMG 120408-PM 4325 cutting insert: (**a**) composition of deposited coating layers; (**b**) macro shot of functional surfaces.

**Figure 2:**
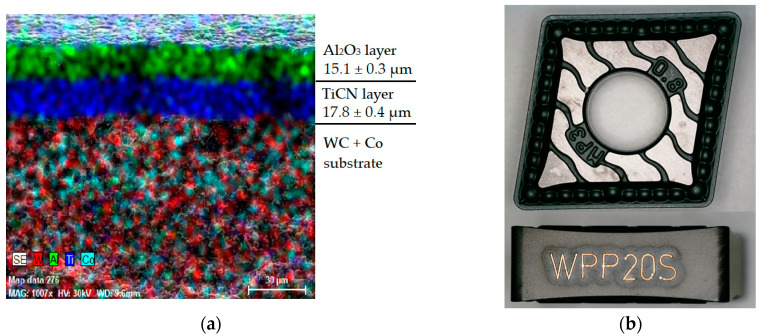
Investigated CNMG 120408E-MP3 WPP20S cutting insert: (**a**) composition of deposited coating layers; (**b**) macro shot of functional surfaces.

The selected indexable cutting inserts were CNMG 120408 type inserts from two different manufacturers. These inserts were purposefully chosen with the same designation according to ISO 1832:2017 standards but with noticeable differences in design, depending on the manufacturer. Specifically, the selected indexable cutting inserts were:

- CNMG 120408-PM 4325: The cutting material designated by the manufacturer as GC4325 (HC) is presented as suitable for finishing to roughing applications when machining steels and cast steels, both in continuous and interrupted cuts, at high metal removal rates. The WC substrate, classified by the manufacturer as P25, is functionally graded using TiCN- and Al_2_O_3_-based coating layers. This was confirmed through elemental mapping of the VBD surface layer in a longitudinal section, as shown in Figure 1a. The chip breaker, labelled by the manufacturer as PM, is designed to allow a wide range of depth of cut (a_p_) and feed rate (f) settings, as seen in Figure 1b. This chip breaker is considered one of the manufacturer’s optimised designs with broad applicability, suitable for both continuous and interrupted cuts. It is recommended not to overload the cutting edge. An EDX analysis confirmed the presence of coating layers with a thickness of 11.5 ± 1.2 µm for the TiCN layer and 5.3 ± 0.3 µm for the Al_2_O_3_ layer.
- CNMG 120408E-MP3 WPP20S: The substrate is specified as a coated cemented carbide with CVD coating layers of TiCN and Al_2_O_3_. According to the manufacturer, the coating layer is optimised with a microstructure of Al_2_O_3_ designed to increase tool life and potentially reduce machining time. The main and secondary flanks are finished to improve wear identification. The EDX analysis of the coating layers confirmed the presence of the declared layers, with a TiCN layer thickness of 17.8 ± 0.4 µm and an Al_2_O_3_ layer thickness of 15.1 ± 0.3 µm, as shown in Figure 2a. The chip breaker labelled as MP3 is primarily intended for medium-duty machining of steel materials that produce long chips. The MP3 geometry is typically used for machining near-net-shape forgings and thinner-walled extruded parts. In the macro image of the VBD in Figure 2b, the so-called “Bullet Design” chip breaker in the shape of a hemisphere is visible, which, according to the manufacturer, should provide additional rigidity for optimal chip breaking.

**Figure 3:**
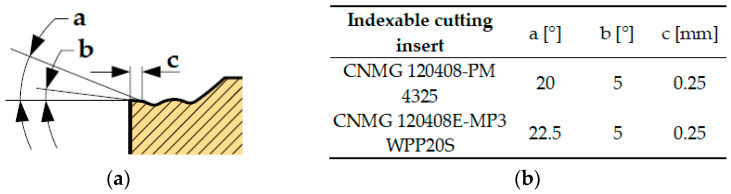
Investigated chamfering structure parameters of evaluated cutting inserts: (**a**) schematic visualisation; (**b**) values for each cutting insert.

Cutting edge parameters that are not identifiable from the marking according to ISO 1832:2017 are declared by the manufacturer and shown in Figure 3. These are chamfering structure parameters, where the rake angle is expressed by value a [°], the bevel of chamfering land is expressed by value b [°] and the land width is expressed by value c [mm].

### 2.2. Experimental Materials

The first experimental material was AISI 4337 steel, a medium-alloyed chromium–molybdenum steel known for its high hardenability, making it suitable for highly stressed machine components. In its quenched and tempered condition, it offers a very favourable strength-to-yield ratio and high toughness. The high toughness of this steel helps to inhibit the propagation of fatigue cracks, resulting in excellent fatigue strength under alternating and combined loading conditions. This material is not susceptible to temper embrittlement. It is typically quenched in oil or synthetic polymer solutions. The chemical composition, as per ASTM A29:2004, is provided in Table 1 [38].

The test material was supplied in the form of 50 mm diameter bars. For the experiment, the material was heat-treated to achieve a hardness of 40–44 HRC to a depth of 15 mm from the surface. The heat treatment consisted of a normalisation at 850 °C, followed by a quenching in oil and a tempering at 450 °C. Hardness measurements were taken from the centre of the workpiece to the surface using a CISAM-ERNST AT250 hardness tester (Cisam–Ernst, Induno Olona, Italy), yielding an average hardness value of 43 ± 1 HRC. The chemical composition of the supplied AISI 4337 material was verified using a Bruker Q4 TASMAN spark spectrometer (Bruker, Billerica, MA, USA). The results of the measurements (see Table 2) confirmed that the supplied material meets the standardised chemical composition requirements. According to the manufacturers of the tested indexable inserts, this material is ideal for machining with these tools.

The second experimental material was AISI 316 L steel, an austenitic stainless steel known for its resistance to pitting corrosion in the presence of chlorides, as well as its good resistance to sulphuric and phosphoric acids. This material is highly weldable without the risk of intergranular corrosion in the heat-affected zone. It is suitable for cold forming and has good machinability, although achieving a mirror finish during polishing can be challenging. AISI 316 L is commonly used in welded structures in aggressive industrial environments, for the manufacture of pressure vessels and for components in the chemical, pharmaceutical and textile industries. Machinability is hindered by the material’s thermal conductivity, higher ductility and tendency to harden during machining. The material’s use in the food industry is influenced by its nickel content. The chemical composition according to ASTM F899:95 is provided in Table 3 [39].

The material of the supplied AISI 316 L semi-finished product, in the form of a 50 mm diameter bar, was tested using a CISAM-ERNST AT250 hardness tester, and an average hardness of 18 ± 0.9 HRC was measured. The chemical composition of the supplied AISI 316 L material was analysed using a Bruker Q4 TASMAN spark spectrometer, and it was found to meet the specified tolerances, as shown in Table 4. According to the manufacturers of the tested cutting inserts, this material does not fall into the primary or secondary usage group for machining purposes.

### 2.3. Experimental Setup

In the framework of the DoE (Design of Experiments), cutting parameters were established for both tested cutting inserts during machining on both test materials. The a_p_ was set to 1.5 mm and the f to 0.3 mm·ot^−1^, which fall within the range of cutting parameters recommended by the manufacturers of both cutting inserts under examination. The cutting speed (v_c_) was set at 100 m·min^−1^, which corresponds to the cutting speeds typically used in conventional machining. The combination of selected cutting parameters was suitable for the given cutting tools and workpiece materials, meeting the requirements for the stability of the cutting process, as demonstrated by initial testing.

The criteria for concluding the experimental phase for each cutting insert was the achievement of a maximum flank wear (VB_max_) of 300 µm. The VB_max_ value was selected for the experiment based on experience with the wear of carbide cutting tools, which typically ranges between 300 and 500 µm [32]. The experimental procedure was as follows: after each tool pass with a_p_ of 1.5 mm, the tested cutting insert was removed and the wear level on the main flank (VB_max_) was measured using an Olympus SZX10 stereomicroscope (Olympus Corporation, Tokyo, Japan) equipped with a Bresser MicroCam-II digital camera (Rhede, Germany). Simultaneously, a sample of the produced chips, corresponding to the achieved flank wear, was collected. To evaluate the shape and size of the chips according to ISO 3685, the Olympus SZX10 stereomicroscope equipped with a Bresser MicroCam-II digital camera and measuring software (ver.: x64, 3.7.13814.20190120) was used again. The chip sample was also evaluated in terms of the number of chips per 100 g of produced chips, providing specific information about the chips set. While the average chip length gives insight into the cutting process itself, the count of chips per 100 g offers information about the quantity and volume of produced chips. This metric, rather than the median chip length, allows for better planning in handling and managing machining waste. Specifically, it may involve sizing waste containers collecting chips, which affects the frequency of waste disposal, movement on the shop floor, storage capacity before transportation for recycling, or even the scheduling of recycling operations like crushing and pressing. A higher chip count indicates the presence of shorter chips overall. After each measurement, the cutting insert was reinstalled in the PCLNR 2020 K12 holder (TaeguTec, Daegu, Republic of Korea), ensuring it returned to its original position. This process was repeated until the specified maximum flank wear (VB_max_ = 300µm) was reached. Each evaluated indexable cutting insert was tested three times to ensure data validity. After reaching the maximum wear limit, the experimental phase was concluded, and the collected data was subsequently analysed, as detailed below.

After reaching the limit wear of the inserts, the roughness of the workpiece was measured. The parameter R_z_ was monitored, which was the average maximum peak to valley of five consecutive sampling lengths within the measuring length. The reason for choosing this roughness parameter was its relevance in the evaluation of surface quality (especially in the automotive industry), and for this reason its numerous uses in practice [40]. A Mitutoyo Surftest SJ-210 portable roughness meter (Mitutoyo, Takatsu Ward, Japan) was used for measurement. The aim of measuring the roughness of the machined surface R_z_ was an informative comparison of the roughness in connection with the achieved limit flank wear of the selected cutting inserts for both test materials.

## 3. Results

**Figure 4:**
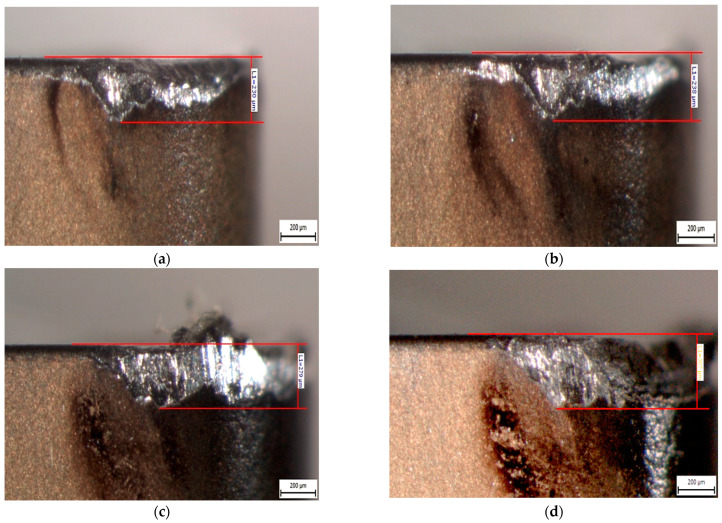
Selected VB_max_ images of CNMG 120408-PM 4325 turning AISI 316 L steel, v_c_ = 100 m·min^−1^: (**a**) T = 2.5 min; (**b**) T = 7.5 min; (**c**) T = 13 min; (**d**) T = 17.5 min.

The obtained data on the tool’s flank wear were plotted against a time axis, where each measured wear value corresponded to a specific tool time in the run T [min]. An example of the continuous measurement of flank wear for a cutting insert is shown in Figure 4, which illustrates the change in the measurement and distribution of flank wear over time.

**Figure 5:**
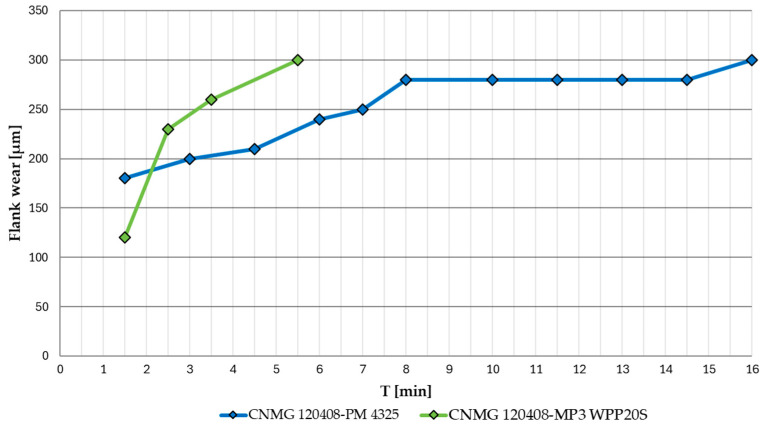
Flank wear progression of evaluated cutting inserts for AISI 4337 steel, v_c_ = 100 m·min^−1^.

Figure 5 shows the wear progression of the two cutting inserts examined during the machining of AISI 4337 steel. The CNMG 120408-PM 4325 insert reached the tool life of the cutting edge, i.e., the specified critical wear at T = 16 [min]. The CNMG 120408-MP3 WPP20S insert reached approximately one-third of the tool life of the cutting edge at T = 5.5 [min]. For both inserts, an initial slowing trend in the wear progression is visible.

**Figure 6:**
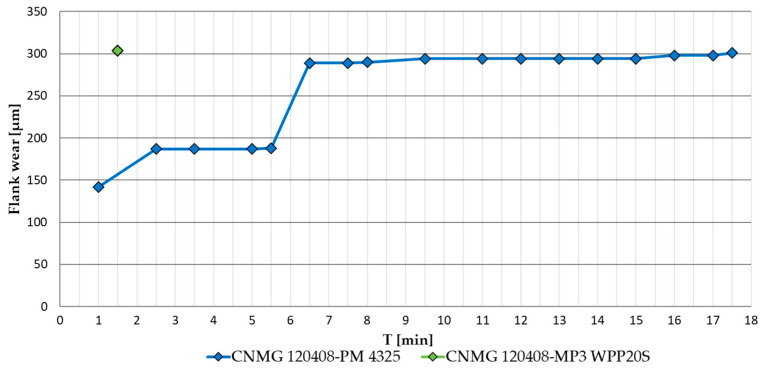
Flank wear progression of evaluated cutting inserts for AISI 316 L steel, v_c_ = 100 m·min^−1^.

The same analysis of the wear progression of the evaluated cutting inserts was conducted using the test material AISI 316 L. Figure 6 shows the wear progression of the CNMG 120408-PM 4325 insert, which reached the specified critical wear at time T = 17.3 [min], which was 1.3 minutes longer than with AISI 4337 material. The CNMG 120408-MP3 WPP20S insert reached critical wear at T = 1.5 [min], practically right after the first cut.

**Figure 7:**
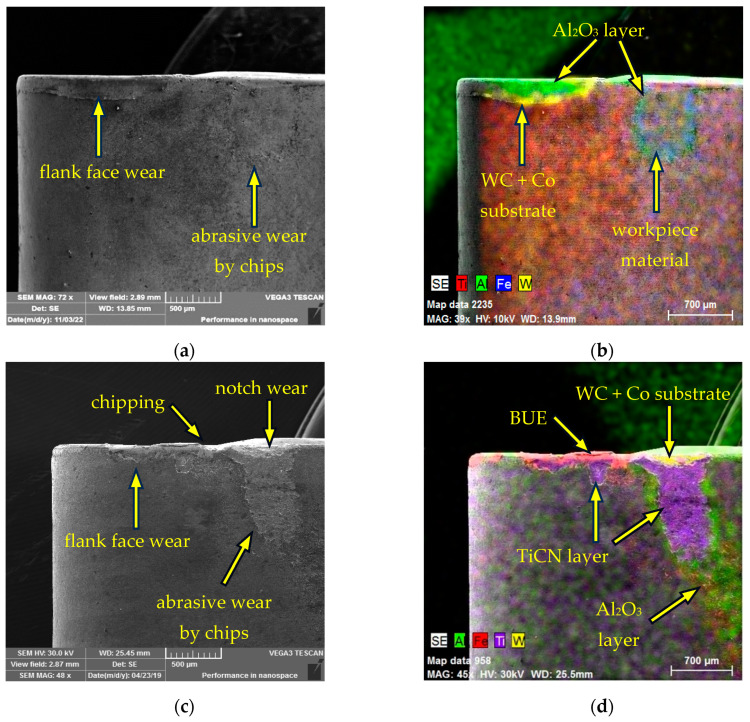
SEM analysis of worn CNMG 120408-PM 4325: (**a**) main flank wear types after turning AISI 4337; (**b**) main flank worn layers after turning AISI 4337; (**c**) main flank wear types after turning AISI 316 L; (**d**) main flank worn layers after turning AISI 316 L.

Using a Tescan Vega 3 SEM (Tescan Orsay Holding a.s., Brno, Czech Republic) equipped with a Bruker X-Flash analyser (Billerica, MA, USA), the cutting inserts under examination that had reached the specified critical flank wear were subjected to a visual assessment of the wear rate of the functional surfaces. Figure 7 shows the flank wear on the CNMG 120408-PM 4325 cutting insert. Figure 7a identifies the forms of flank face wear after machining AISI 4337 steel. The flank face surface was abrasively worn by chips in the area of chip exit from the cutting point. In Figure 7b, this worn condition is subjected to elemental (EDX) analysis, which confirms the presence of an Al_2_O_3_ layer in the flank face wear area down to the level of the support substrate. A portion of the workpiece material adhered in the chip exit area, revealing the Al_2_O_3_ layer to a small extent. Similarly, a visual assessment of the wear of the CNMG 120408-PM 4325 insert after machining AISI 316 L is shown in Figure 7c, where several types of wear were identified, namely flank face wear, abrasive wear by chips, chipping at the cutting edge and notch wear at the interface between the cutting tool edge and the workpiece surface. Figure 7d shows the wear at the notch wear point down to the level of the substrate, highlighting the wear limits of the TiCN and Al_2_O_3_ coatings. Additionally, a built-up edge (BUE) formed by the machined AISI 316 L material is visible. Overall, the wear rate was higher after machining AISI 316 L. Although similar flank face wear was achieved, the wear boundaries of each coating layer were more obvious after machining AISI 316 L stainless steel and there was more intense wear of the cutting edge in the form of chipping compared to machining AISI 4337.

**Figure 8:**
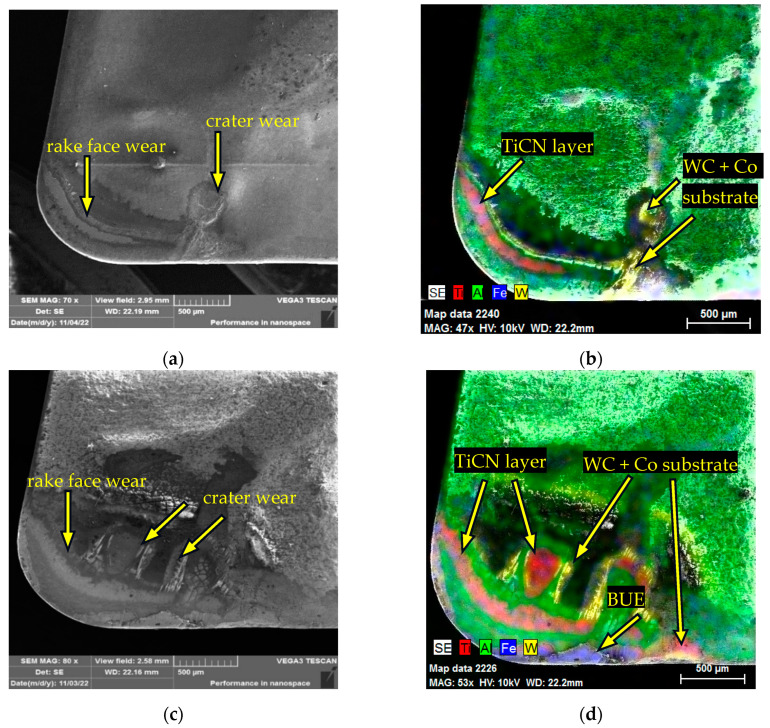
SEM analysis of worn CNMG 120408-PM 4325: (**a**) main rake wear types after turning AISI 4337; (**b**) main rake face worn layers after turning AISI 4337; (**c**) main rake wear types after turning AISI 316 L; (**d**) main rake face worn layers after turning AISI 316 L.

The identification of the types of wear and the degree of exposure of the individual coating layers, and possibly the substrate, on the face of the CNMG 120408-PM 4325 insert after reaching the critical wear limit are shown in Figure 7 for AISI 4337 steel. Beyond the rake face wear line that follows the edge of the cutting insert, wear in the form of crater wear was visible at the interface between the cutting edge of the insert and the workpiece edge, as shown in Figure 8a. As part of the experiment, a crater wear width (KB) of 785.7 ± 33.7 µm was measured on CNMG 120408-PM 4325 after reaching the wear limit VB_max_ when machining AISI 4337. In Figure 8b, wear is noticeable at the level of the TiCN coating on the rake face wear area and wear down to the level of the support substrate below the crater wear area. Figure 8c shows a picture of the worn sheet of rake face after turning AISI 316 L steel. A rake face wear line can be seen following the radius of the tip of the insert. Crater wear is scattered across the rake face. On CNMG 120408-PM 4325, KB = 709.2 ± 28.3 µm was measured after reaching the wear limit VB_max_ when machining AISI 316L. Within the rake face wear and crater wear, both the TiCN layer and the supporting substrate were exposed, which is visible in Figure 8d. Also, the workpiece material was identified in the form of BUE and notch wear on the cutting edge, which was also evident on the rake face. Similar to the main flank face, the rake face in the case of AISI 316 L machining experienced more intense surface wear and also more of the machined material was adhered to the face and the cutting edge.

**Figure 9:**
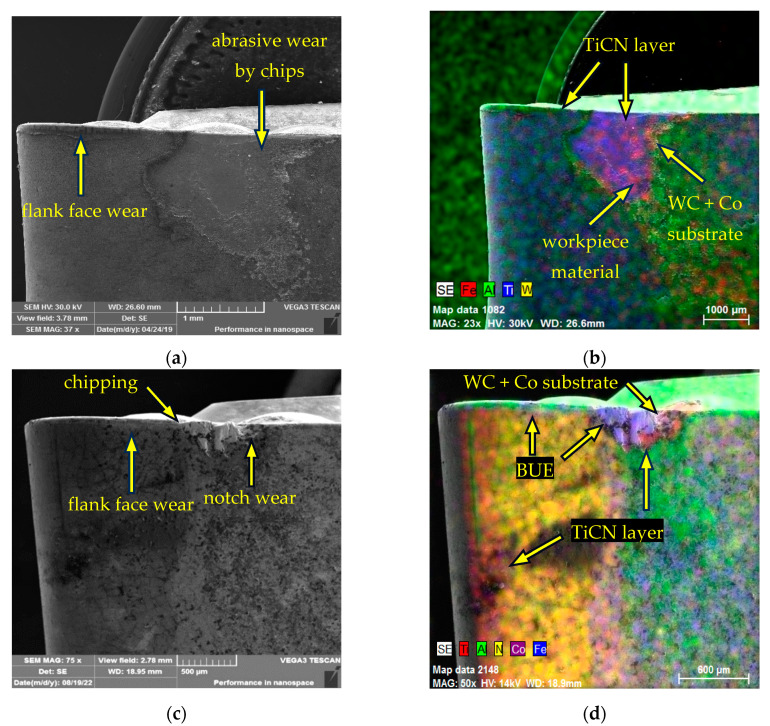
SEM analysis of worn CNMG 120408-MP3 WPP20S: (**a**) main flank wear types after turning AISI 4337; (**b**) main flank worn layers after turning AISI 4337; (**c**) main flank wear types after turning AISI 316 L; (**d**) main flank worn layers after turning AISI 316 L.

The wear patterns of the main flank face of the CNMG 120408-MP3 WPP20S insert after machining of AISI 4337 steel and the elemental analysis of the worn surfaces are shown in Figure 9a,b. The flank face wear and abrasive wear by chips are clearly visible and are defined with an exposed TiCN layer, locally adhered workpiece material (chips) and even partially exposed support substrate. The wear rate is therefore higher than in the case of flank face wear. In Figure 9c, after the machining of ASIS 316 L steel, chipping, flank face wear and notch wear can be seen at the interface between the cutting edge and the workpiece surface. In Figure 9d, EDX analysis confirms a more intense level of wear at the level of the TiCN layer in the area of the tip of the tool, and the substrate level in the notch wear area, which was partially filled by the workpiece material in the form of BUE. BUE is mainly observed at the areas where the underlying coating layers or substrate material have been exposed. Again, after machining AISI 316 L, the BUE was more extensive due to more intense wear at the level of the substrate.

**Figure 10:**
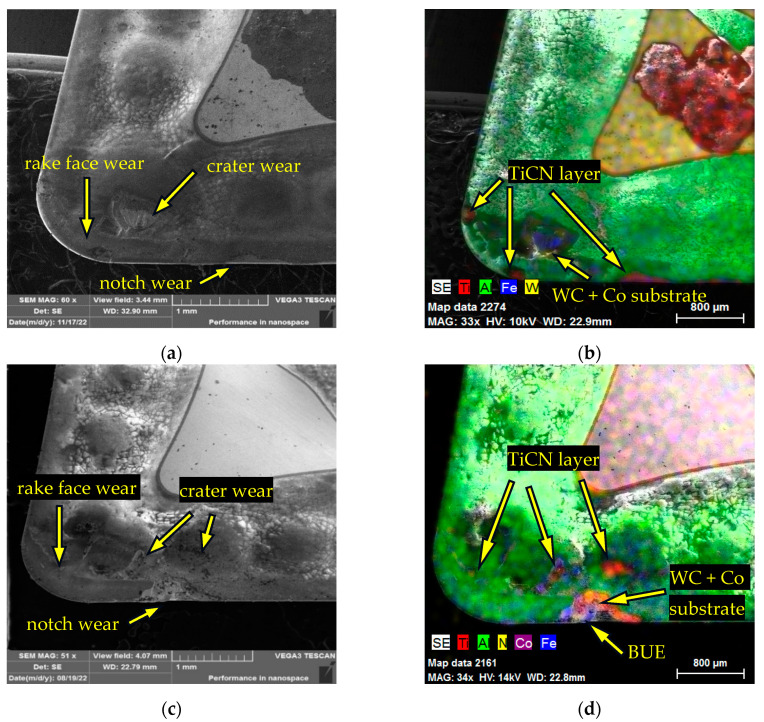
SEM analysis of worn CNMG 120408-MP3 WPP20S: (**a**) main rake wear types after turning AISI 4337; (**b**) main rake face worn layers after turning AISI 4337; (**c**) main rake wear types after turning AISI 316 L; (**d**) main rake face worn layers after turning AISI 316 L.

The wear patterns on the rake face of CNMG 120408-MP3 WPP20S after turning AISI 4337 steel are identified in Figure 10a. Notable observations include rake face wear and crater wear on the main surface near the chip formers in the form of hemispheres. As part of the experiment, KB = 726 ± 37.3 µm was measured on CNMG 120408-PM3 WPP20S after reaching the wear limit VB_max_ when machining AISI 4337. Notch wear was visible at the interface between the insert cutting edge and the workpiece. The EDX analysis in Figure 10b shows the extent of wear at the local level of the TiCN layer along the line of the cutting edge and the WC + Co substrate at the place where the crater wear was formed. In Figure 10c, the wear in the form of rake face wear, crater wear and notch wear after turning AISI 316 L is shown. On CNMG 120408-PM3 WPP20S, KB = 648.1 ± 36.1 µm was measured after reaching the wear limit VB_max_ when machining AISI 316L. EDX analysis depicted in Figure 10d identifies the exposure of the TiCN layer along the boundaries of the crater wear area. Additionally, the wear along the crater wear boundaries has reached the substrate level, which is visible even in the notch wear area. The adhered material could be observed both at the notch wear area and along the crater wear boundaries. Identical types of wear were identified on the rake face, but with less intensity or distribution in the case of AISI 4337 machining compared to AISI 316 L machining.

**Figure 11:**
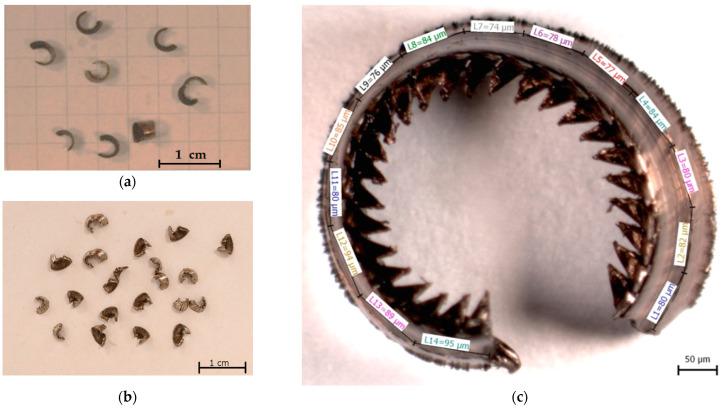
Demonstration of macroscopic chip evaluation: (**a**) CNMG 120408-PM 4325 chips after machining AISI 4337, T = 13 min; (**b**) CNMG 120408-PM 4325 chips after machining AISI 316 L, T = 17.5 min; (**c**) example of micrometric chip length measurement.

The experiment also included an analysis of the produced chips in terms of their length and shape. The resulting chronologically arranged photographic documentation of the chips provided the basis for the assessment of the shape of the chips (see Figure 11a,b), and also for the measurement of the length of the chips, which was carried out using the micrometric method (see Figure 11c). As part of the shape assessment, 5 to 15 chips were assessed per chip collection for each workpiece material removal. Length measurements were made on 3 to 5 pieces per each chip collection.

The analysis results of the produced chips are presented in Table 5. For both tested inserts, the data are divided according to the test material. The actual evaluation consisted of an assessment of chip shape/shapes, average length and average number of chips per 100 g of chips produced. From the measured data, it can be seen that despite different designs of the chip formers on the evaluated inserts, the same chip shapes (arc loose and arc connected) were identified, and their average length did not differ significantly within the overall production. A noticeable difference was found in the average number of chips per 100 g of chips. For both cutting inserts tested, this parameter was more favourable when machining AISI 4337 steel. In the case of the CNMG 120408-MP3 WPP20S insert, the difference in chip production between the tested materials was greater, and it achieved the best results in terms of chip production. When comparing the tested inserts after machining AISI 316 L, the results in terms of chip production were practically identical.

The measured values of the roughness parameter R_z_ on the surface of the machined test blank after reaching the flank wear limit are shown in Table 6, where a better result is evident for the insert CNMG 120408-PM 4325 compared to CNMG 120408E-MP3 WPP20S.

## 4. Discussion

Based on the analysed data, the wear process of the examined cutting inserts can be summarised as follows: When machining AISI 4337 steel, the CNMG 120408-PM 4325 insert achieved a longer tool life of 16 minutes, nearly three times that of the CNMG 120408E-MP3 WPP20S insert, which reached its critical wear after just 5.5 minutes. This difference may be attributed to the specific design of the CNMG 120408E-MP3 WPP20S insert, despite its classification under the same ISO 1832:2017 standard as the CNMG 120408-PM 4325. The main differences include the longitudinally curved cutting edge and the hemispherical chip breaker on the CNMG 120408E-MP3 WPP20S. The types of wear observed on the flank and rake faces of the tools are generally similar, with the exception of the notch wear observed on the rake face of the CNMG 120408E-MP3 WPP20S. A noticeable difference is in the wear of the coating layers, which are more clearly defined with distinct boundaries on the flank face of the CNMG 120408E-MP3 WPP20S. Conversely, the rake face of the CNMG 120408-PM 4325 insert exhibited more intense wear. However, in terms of dimensional accuracy of the workpiece, surface roughness and the potential introduction of stress into the surface layer, the monitoring of the main flank wear of the cutting tool is preferred.

The reason for the greater wear of the coating layer on the CNMG 120408E-MP3 WPP20S insert when machining AISI 4337 might paradoxically be due to the fact that this insert has coating layers of Al_2_O_3_ and TiCN that are up to twice as thick as those on the CNMG 120408-PM 4325 insert. This increased thickness could result in larger sections of the coating being detached, particularly in scenarios where the chip exits the cutting zone (causing abrasion against the tool’s flank) and where there is a point contact between the workpiece and the cutting tool at the interface between the workpiece surface and the tool’s cutting edge. It is possible that with thicker coatings, the layer composition may not adhere as well to the substrate, or the thicker layer might be subjected to greater stress from the cutting force during machining. This could mean that the coating used on the CNMG 120408E-MP3 WPP20S insert is less suitable for the specific combination of cutting conditions and workpiece material [41]. To verify this hypothesis, it is advisable to conduct a visual analysis of the cutting process using a high-speed camera focused on the main flank area of the tool.

When machining AISI 316 L steel, the CNMG 120408-PM 4325 insert again demonstrated superior tool life, lasting 17.5 minutes compared to the CNMG 120408E-MP3 WPP20S insert, which reached its critical wear limit on the main flank after just 1.5 minutes, immediately after removing the first layer of AISI 316 L material. This clearly shows that the CNMG 120408E-MP3 WPP20S insert is not suitable for machining austenitic stainless steel. Austenitic stainless steels are tough materials with poor thermal conductivity, requiring more energy for cutting. Consequently, a smaller proportion of the heat generated during machining is carried away by the chips, and more of this thermal energy is transferred through the tool itself. This can accelerate the wear process. The rapid wear observed when using the CNMG 120408E-MP3 WPP20S insert, despite the expectation that its thicker coating layers would provide greater durability when machining AISI 316 L, showed the exact opposite trend. The cause of this wear pattern could be attributed to the tool’s geometry or the spherical shape of the chip breaker. On the other hand, the CNMG 120408-PM 4325 insert achieved a longer service life by 1.5 minutes when machining AISI 316 L, a material that is neither the primary nor an alternative recommendation for machining by the manufacturer. This outcome exceeded both theoretical and practical expectations, as it performed better in AISI 316 L machining than in the machining of hardened AISI 4337 steel.

The wear rate of the examined cutting inserts after machining AISI 316 L steel was more intense, exhibiting phenomena typically associated with the machining of austenitic steels, such as a higher degree of material adhesion in the form of built-up edge (BUE). On the CNMG 120408-PM 4325 insert, a greater degree of coating layer wear was observed on the flank, particularly at the interface between the machined material and the cutting edge, including more pronounced notch wear that exposed the substrate. Chipping of the cutting edge was also noted, along with more severe crater wear and the presence of adhered workpiece material in the form of BUE on the rake face. Through a combination of high cutting temperature and abrasion, the outgoing chips cause wear of the coating layers on the rake face and subsequently release the tungsten carbide grains from the basic structure of the substrate. As a result of diffusion, carbon leeches into the chips and thus the crater on the rake face increases. This mechanism is more intensive in the case of machining hard materials [42]. This explains the approximately 80 µm higher KB values when machining hardened steel AISI 4337. On the contrary, a larger layer of Al2O3 coating, which protects against the effect of the cutting temperature, in this case ensured a smaller crater wear on CNMG 120408-PM3 WPP20S [43]. However, this positive effect was observed only in the case of the formation of crater wear. The observed wear forms and their distribution correspond to the process of machining austenitic stainless steels and the previously described mechanisms of heat distribution that occur during the machining of this type of material. The rapid wear of the CNMG 120408E-MP3 WPP20S insert further underscores its unsuitability for applications involving austenitic stainless steels. A significant notch wear developed at the interface between the cutting edge and the workpiece surface, where material buildup in the form of BUE was evident. This occurrence is expected, as the freshly exposed layers of the cutting tool and the machined material surface in the form of chips are chemically clean. The formation of BUE on these surfaces is promoted by a combination of cutting temperature, pressure and the excellent adhesion properties of these chemically clean surfaces.

The process of continuous chip evaluation involved analysing their length and calculating the number of chips per 100 grams of produced chips. The outcome of processing these data was the average chip length and the average number of chips per 100 grams of produced chips for each cutting insert and machined material. In terms of average chip length, no significant differences were observed under the specified cutting conditions, either between the examined cutting inserts or between the machined materials, AISI 4337 and AISI 316 L. This fact contributes positively to the evaluation of chip formation by the examined cutting inserts within the application of the selected cutting conditions and machined materials, which differ significantly in terms of temperature distribution [44]. One would expect the average chip length to vary more as longer chips are formed during the machining of austenitic steels due to the usual higher ductility. This is also related to the austenitic structure of the machined material and the distribution of heat to the cutting tool, which creates chips of irregular shape. In contrast, shorter-length chips were expected to form during the machining of hardened steel [44,45]. Due to the cutting speed v_c_ = 100 m·min^−1^, it is possible that during the machining of austenitic steel, adhesive wear was applied to a greater extent, which enabled the transfer of a larger amount of heat to the cutting insert, which could change the morphology of the formed chips [46]. From a production planning perspective, it is beneficial that the evaluated average lengths of the formed chips show relatively small deviations.

For the purposes of technological planning, the chips produced were also evaluated based on the number of chips generated per 100 grams of chips. This evaluation method provides a more detailed specification of the chips produced, taking into account their physical shape beyond just their average length, which is crucial for planning waste management. Similar to the average chip length, no significant differences in the number of chips were observed, except in the case of the CNMG 120408-E MP3 WPP20S cutting insert when machining AISI 4337 steel. In this scenario, the number of chips was 3831 ± 691 per 100 grams of chips, which is approximately 1000 more chips compared to the other combinations of cutting inserts and workpiece materials. This represents the best result concerning this parameter. The other combinations were comparably consistent. It can thus be stated that the designed spherical ‘Bullet’ chip breaker of the CNMG 120408-E MP3 WPP20S insert effectively shaped the chips in terms of their suitability in form and length under the given cutting conditions, particularly when machining hardened AISI 4337 steel. Given the primary purpose of the CNMG 120408-E MP3 WPP20S insert, as described in Chapter 2.1, this outcome was anticipated. In contrast, the CNMG 120408-PM 4325 insert, which achieved excellent durability results in the experiment on both tested materials, showed similar results regarding the number of chips per 100 grams across both test materials. This suggests that the CNMG 120408-PM 4325 insert is a more versatile cutting tool design in terms of its application.

As an informative addition, the roughness parameter R_z_ of the machined surface was measured after reaching the critical flank wear. The measured values showed a certain correlation with the achieved service life of the inserts when applied to specific test materials. Better results were achieved by the CNMG 120408-PM 4325 insert, with even better R_z_ values achieved by this insert when machining AISI 316 L. It is likely that similar mechanisms were involved in the formation of the machined surface profile as in chip formation in the results observed in this experiment [45]. There is an obvious connection between the roughness of the machined surface, the morphology of the formed chip and the wear mechanism of the cutting tool based on the cutting temperature, influenced by factors such as the properties and composition of the machined material and cutting conditions, especially the cutting speed [45,47]. For more complex results, it is recommended to analyse other components of surface integrity in connection with the wear of cutting tools.

## 5. Conclusions

CNMG 120408-coated indexable cutting inserts from two different manufacturers under identical cutting conditions were compared. As test materials, hardened steel AISI 4337, primarily intended for the examined cutting inserts, and stainless steel AISI 316L, which is not intended for use with the examined cutting inserts, were used. The course and character of the main flank wear were monitored until the set criterion VB_max_ = 300 µm was reached. At the same time, the produced chips were continuously assessed in terms of their shape, length and number of chips per 100g of chips produced according to ISO 3685. The results of the mutual comparison of indexable cutting inserts can be summarised in the following points:

- During wear testing during the machining of hardened AISI 4337 steel, which fell into the primary application group of the tested inserts, the CNMG 120408-PM 4325 insert achieved a life of T = 16 min, which was almost three times longer than the CNMG 120408E-MP3 WPP20S insert, which reached a service life of T = 5.5 min during the machining of hardened steel.
- During wear testing during the machining of AISI 316 L stainless steel, which according to the manufacturers of the examined inserts is not suitable for the application of the tested tools, the CNMG 120408-PM 4325 insert achieved a life of T = 17.3 min. The CNMG 120408E-MP3 WPP20S insert reached critical wear during machining of AISI 316L already at T = 1.5 min.
- The analysis of coating layers showed that CNMG 120408E-MP3 WPP20S has thicker layers of Al2O3 and TiCN coatings applied compared to CNMG 120408-PM 4325. Despite this, it achieved significantly worse durability results in the experiment and proved to be completely unsuitable during the machining of AISI 316L.
- The results of the evaluation of the morphology of the worn surface of the examined inserts indicate that a greater applied thickness of the coatings can reduce the occurrence of some types of wear (e.g., chipping), but it is not a guarantee that it will increase the overall tool life of the functional parts of the cutting tool within the application. Paradoxically, a lower thickness of the coating protection could be more advantageous from the point of view of reducing the risk of its separation even in the case of machining a material that does not dissipate heat well; see AISI 316 L described above.
- The shape and average length of the produced chips did not differ practically within the machining of both test materials. Despite the different designs of the chip formers, the evaluated inserts from both manufacturers showed similar results.
- As part of the evaluation of the number of chips per 100g of chips produced, CNMG 120408E-MP3 WPP20S achieved a better result during the machining of AISI 4337, when the number of chips produced per 100g of chips was set at 3831 ± 691 pcs, compared to CNMG 120408-PM 4325, when this figure was equal to 2782 ± 350. During the machining of AISI 316 L, the results of this parameter were practically identical for both evaluated inserts.
- It is possible that the so-called ‘Bullet design’ is more advantageous from the point of view of chip formation when applied to noble low to medium alloy steels; however, when applied to austenitic stainless steels, the importance decreases.
- Despite the identical marking of interchangeable inserts according to ISO 1832:2017 and the available parameters provided by the manufacturer, such as the composition of the coating layers or the basic geometry of the edge with the shape of the chip breaker, the differences in the functional properties of the cutting tools under the given conditions are striking, which increases the importance of choosing a suitable specific cutting tool.
- A connection was observed between the measured roughness parameter Rz of the machined surface, the morphology of the formed chip and the wear mechanism of the cutting tool with respect to the measured values after reaching the flank wear limit.

## Figures and Tables

**Table 1:** Chemical composition of AISI 4337 according to ASTM A29:2004 (in weight %).

C	Mn	P	Cr	Mo	S	Ni	Si
0.3–0.38	0.5–0.8	< 0.4	1.3–1.7	1.3–1.7	0.15–0.3	<0.035	<0.035

**Table 2:** Chemical composition of AISI 4337 according to Bruker Q4 Tasman (in weight %).

C	Mn	P	Cr	Mo	S	Ni	Si
0.342	0.57	0.329	1.519	1.47	0.193	<0.005	<0.001

**Table 3:** Chemical composition of AISI 316 L according to ASTM F899:95 (in weight %).

C	Mn	P	Cr	Mo	S	Ni	Si
<0.03	<2	<0.045	16.5–18.5	2–2.5	<0.015	10–13	<1

**Table 4:** Chemical composition of AISI 316 L according to Bruker Q4 Tasman (in weight %).

C	Mn	P	Cr	Mo	S	Ni	Si
0.022	1.407	0.041	17.24	2.007	0.007	10.6	0.578

**Table 5:** Comparison of produced chips for individual cutting inserts and materials.

	CNMG 120408-PM 4325		CNMG 120408E-MP3 WPP20S	
	AISI 4337	AISI 316 L	AISI 4337	AISI 316 L
Chip Shape	Arc loose Arc connected	Arc loose Arc connected	Arc loose Arc connected	Arc loose Arc connected
Average Length [mm]	7.5 ± 0.6	6.2 ± 0.9	6.5 ± 1.3	6.3 ± 0.6
Avg. Number of Chips per 100 g of Chips [pcs]	2782 ± 350	2569 ± 243	3831 ± 691	2503 ± 408

**Table 6:** Comparison of R_z_ parameter for individual cutting inserts and materials after reaching the flank wear limit.

Roughness Parameter	CNMG 120408-PM 4325		CNMG 120408E-MP3 WPP20S	
	AISI 4337	AISI 316 L	AISI 4337	AISI 316 L
R_z_ [µm]	12.45 ± 0.229	10.017 ± 0.765	14.975 ± 0.259	15.722 ± 0.346

## Data Availability

The original contributions presented in the study are included in the article, further inquiries can be directed to the corresponding author.
